# What makes or breaks a campaign to stop an invading plant pathogen?

**DOI:** 10.1371/journal.pcbi.1007570

**Published:** 2020-02-06

**Authors:** Alice E. Milne, Tim Gottwald, Stephen R. Parnell, Vasthi Alonso Chavez, Frank van den Bosch

**Affiliations:** 1 Sustainable Agricultural Systems, Rothamsted Research, Harpenden, United Kingdom; 2 USDA-ARS Fort Pierce, Florida, United States of America; 3 Ecosystems and Environment Research Centre, School of Science, Engineering and Environment, University of Salford, Greater Manchester, United Kingdom; 4 Department of Environment and Agriculture, Centre for Crop and Disease Management, Curtin University, Perth, Australia; University of California, Los Angeles, UNITED STATES

## Abstract

Diseases in humans, animals and plants remain an important challenge in our society. Effective control of invasive pathogens often requires coordinated concerted action of a large group of stakeholders. Both epidemiological and human behavioural factors influence the outcome of a disease control campaign. In mathematical models that are frequently used to guide such campaigns, human behaviour is often ill-represented, if at all. Existing models of human, animal and plant disease that do incorporate participation or compliance are often driven by pay-offs or direct observations of the disease state. It is however very well known that opinion is an important driving factor of human decision making. Here we consider the case study of Citrus Huanglongbing disease (HLB), which is an acute bacterial disease that threatens the sustainability of citrus production across the world. We show how by coupling an epidemiological model of this invasive disease with an opinion dynamics model we are able to answer the question: What makes or breaks the effectiveness of a disease control campaign? Frequent contact between stakeholders and advisors is shown to increase the probability of successful control. More surprisingly, we show that informing stakeholders about the effectiveness of control methods is of much greater importance than prematurely increasing their perceptions of the risk of infection. We discuss the overarching consequences of this finding and the effect on human as well as plant disease epidemics.

## Author summary

The successful regional control of emerging and invasive diseases often requires that a sufficiently large proportion of the population comply with the control strategy. This is notably the case in diseases such as measles but also applies to epidemics in animals and plants. If insufficient numbers of stakeholders comply with disease control, or if control becomes uncoordinated for some reason, then control fails. Therefore, both epidemiological and human behavioural factors influence the outcome of emerging, endemic, and invasive disease control campaigns. Mathematical models are often used to determine factors that are important for disease control to be successful, but these models tend to focus on the epidemiology and efficacy of control, frequently neglecting human behaviour. A number of mathematical models of human disease, and to some extent animal disease do incorporate participation or compliance behaviours; however, studies looking at human actions and attitudes towards plant disease control are quite rare and almost exclusively driven by pay-offs or direct observations of the disease state. It is however very well known that opinion, for example about how effective control is perceived to be, is also a key driving factor of human decision making. Here we consider the case study of Citrus Huanglongbing disease (HLB), a devastating invasive disease in citrus which threatens production worldwide. We show how by coupling an epidemiological model with an opinion dynamics model it is possible to answer the question: What makes or breaks the effectiveness of a disease control campaign?

## Introduction

The control of agricultural pests and diseases is often most effective if the treatment is applied in a coordinated way across a region. This is particularly true in cases where growers cannot protect their crops in isolation but rely on the cooperation of others to achieve eradication or suppression of the pest or disease in their area. There have been many studies that look at the population dynamics of plants and their pathogens to determine optimal approaches for control of such pests and diseases [1–3], but none of these have accounted for the fact that effective control often relies on the voluntary assimilation of the control methods by decision makers, i.e. growers. These individuals must weigh the relative costs, risk of infection, and the reliability of control methods. If individuals do not co-operate then control is almost certain to fail. Control success therefore relies not just on the efficacy of the available methods of control, but also on factors related to human behaviour. This leads us to the question: *What makes or breaks an effective disease control campaign*? Our question applies to many plant-pathogen systems from around the globe, each with its own unique set of epidemiological, economic and social constraints. Here we consider, arguably one of the most serious of these, Huanglongbing disease (HLB), also known as citrus greening.

Huanglongbing (HLB) is a devastating disease in citrus which threatens the sustainability of citrus production throughout the world and has caused billions of dollars’ worth of loses [4]. For example, in Florida, the disease was first found in 2005 and has since caused more than an 80% reduction in citrus production [5,6]. It is now considered unlikely that the Florida citrus industry will survive in its current form. In 2012 the disease was found for the first time in California, and since that time over 1500 trees have been confirmed to be infected [7]. The industry, therefore, is in desperate need for guidance on the development and deployment of effective control methods.

**Fig 1:**
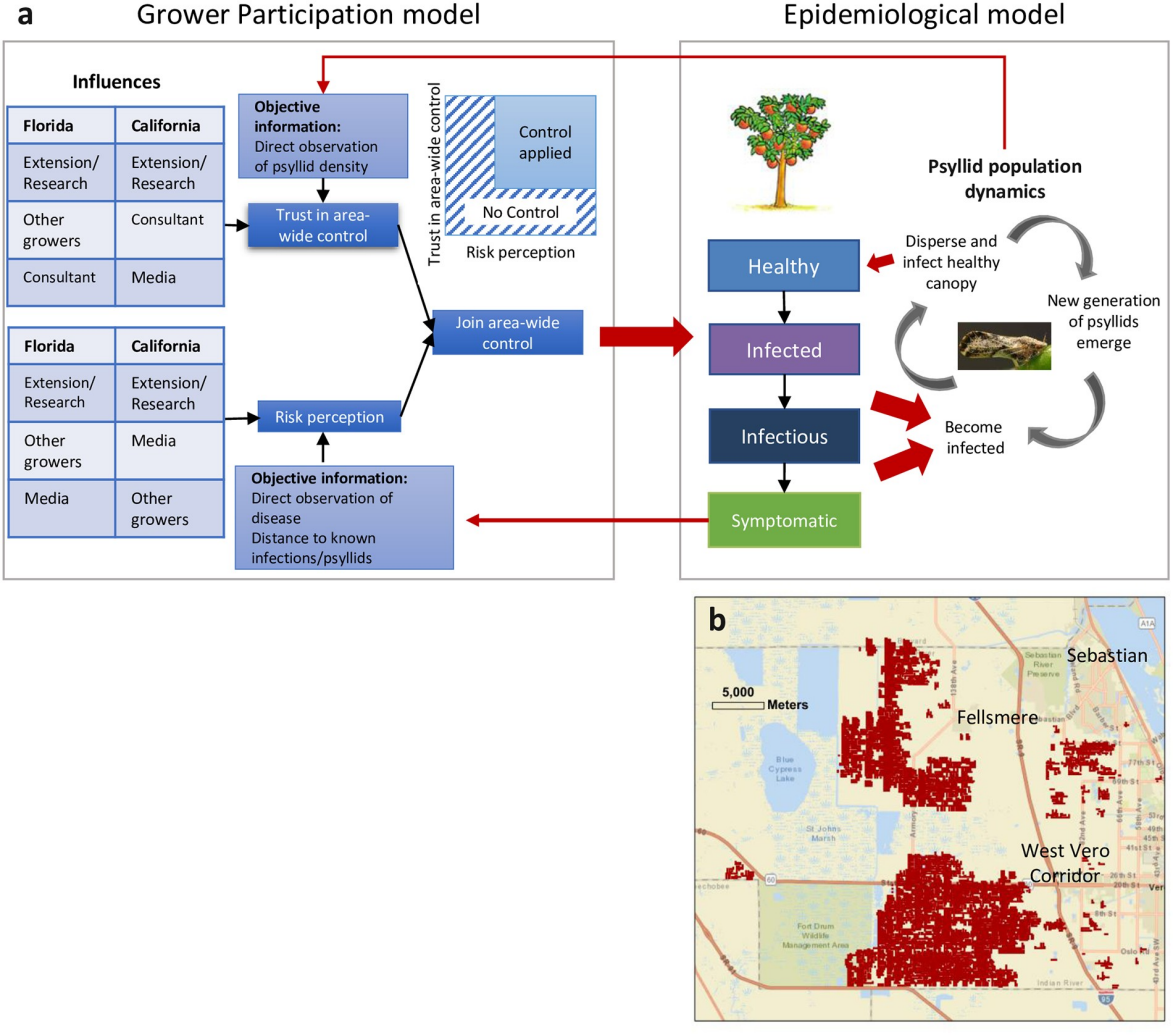
A model of HLB in the landscape. (a) A schematic showing how the grower participation model is linked to the epidemiological model. Growers join an area-wide control program if their risk perception and trust in area-wide control are high. This impacts the psyllid population and so the dynamics of the disease in the landscape. Observations of infection increase risk perception and can erode the trust in area-wide control. Red arrows indicate where models interact. (b) A simulated landscape representing a typical Citrus Health Management Area in Florida. The area where commercial citrus is grown is indicated by red shading.

Huanglongbing (HLB) disease is caused by a fastidious bacterium, *Candidatus* liberibacter spp., with three species known to be associated with HLB symptoms in different regions. In the USA, the Asian Citrus Psyllid (ACP, *Diaphorina citri*) is the invasive vector that is responsible for spreading *Candidatus* Liberibacter asiaticus (CLas) [4]. A healthy citrus tree may become infected with CLas when a psyllid carrying the bacterium, i.e. bacterialiferous, feeds on that tree (Fig 1a). After infection, each newly established CLas population increases and begins to spread from the inoculation point non-uniformly within the tree. Post-infection, the tree enters a cryptic period after which the tree becomes infectious allowing psyllids to acquire the bacterium, become bacterialiferous, and become capable of spreading the pathogen. Eventually, after a period of a few weeks to multiple months the tree becomes symptomatic. The psyllids fitness is positively affected by the acquisition of the bacterium with slightly increased fecundity [8]. Psyllids fly directionally over short distances to neighbouring groves but over longer distances are susceptible to prevailing air currents [9].

Currently, there is no known cure for HLB and so control relies largely on controlling the psyllid by spraying insecticide, removing inoculum sources (infected trees), and ensuring clean plant material for planting. However, because these insects can travel long distances, growers cannot protect themselves from the disease by spraying their orchards in isolation, they are reliant on their neighbours controlling as well. Experts around the world therefore advocate the use of ‘area-wide control’ whereby individual growers in an area coordinate their spray applications over a large area [10]. Other control methods are generally considered less effective [4,10,11].

To understand how to successfully control HLB across a region we must therefore look at both epidemiological factors and the social factors that motivate growers’ choices on control. Historically, human behaviour has been considered as an important factor in the spread of infectious diseases, becoming a reasonably well studied topic in human, and to some extent, animal diseases [12–17]. Models on the interplay between human behaviour and disease epidemics have been developed on the basis of direct observations of the disease state [14,16,18], contact networks [19–22], mean-field with global mixing between individuals in the population [14] and spatial cellular automata models [23] to mention some. Previous work on the interplay between human behaviour and the spread of animal and human diseases have largely assumed that decisions are based on cost-benefit [14,24]. However, it is well known that opinion and perception are important driving factors of human decision making [25–27]. Therefore we chose to use Opinion Dynamics [28–32] to develop a model of grower behaviour that we couple with a spatially explicit model of the regional dynamics of HLB. A similar approach has been used to describe the dynamics of avian flu in humans [33]. The model of the spread of HLB uses an abundance-based population model to describe the life-cycle of ACP with dispersal modelled stochastically through a landscape. The transfer of infection from ACP to trees results in healthy trees becoming infected and then passing through latent (infected but not yet infectious), cryptic (infectious) and symptomatic stages (symptomatic and infectious). Once infectious, trees are able to pass on infection to non-bacterialiferous ACP (Fig 1). In the examples we consider, the citrus tree population is structured in plantings of orchard blocks that are arranged in a spatial pattern in the landscape (Fig 1b).

Before developing the opinion dynamic model, we surveyed growers in Florida and California to find out what the key drivers are for a grower to decide to join an area-wide control campaign [7]. Fig 1a shows the conceptual model developed on the basis of this survey, where the two key drivers are the risk perception (quantified as a grower’s perceived probability that their grove will become infected) and the trust in control (quantified by a grower’s perceived probability that area-wide control is effective). These factors accord with those reported to affect the public’s adoption of prevention measures for human diseases that are known to be difficult to cure [34]. These opinions are influenced by other growers, consultants, extension workers and researchers and to a lesser extent by the media [7]. Direct observations also play an important role in opinion. Firstly, the observed state of the epidemic, for example by neighbouring plantings becoming infected, increases the risk perception of the grower considerably. Secondly, when a grower applies the control and subsequently his plantings become infected, the trust in the control method decreases considerably. When the perceived risk of infection, as well as the trust in the control options are both high, a grower is inclined to join an area-wide control scheme. We did not consider the importance of the economics of crop production and disease control in our model. By not including the economics associated with control we simplified our model so allowing a clearer analysis of the opinion dynamics factors that affect the success or failure of a disease control campaign.

The epidemiological and the opinion dynamic model are coupled by (i) the direct observations growers make on the development of the epidemic, affecting their opinions on risk and trust in control, and (ii) growers joining or not joining the area-wide-control scheme that affects the course of the epidemic (Fig 1a). We determine which of the factors in the models of behaviour and control efficacy are most important for effective disease control.

## Methods

### The epidemiological model

We developed a model of the spread of HLB in citrus orchards across an area typical of a Citrus Health Management Area (CHMA) in Florida [7]. Several models for the epidemiology of HLB have been developed and tested [35–38], and our model is a variant of these. The modelled CHMAs were based on USDA statistics which describe the locations of citrus orchard blocks and their areas. The citrus tree population is structured in plantings of orchard blocks that are arranged in a spatial pattern in the landscape (Fig 1b). We modelled each CHMA with a grid of cells, with each cell representing 1 ha of land. We approximated each block area to the nearest ha and located the associated number of cells around the centroid for this area. This resulted in realistic distributions of citrus across our modelled CHMAs (see Fig 1b). For one CHMA (Indian River County) we had data on the ownership of the orchard blocks (for anonymity purposes each owner was replaced by a numeric reference number). This allowed us to identify blocks that were assumed to be managed by the same decision maker (i.e. grower). For other CHMAs we modelled, we used the distribution of block-sizes per grower from Indian River, as this is the only CHMA for which we have such data, to stochastically assign block sizes (i.e. numbers of cells) to the growers. The cell allocation was arranged so that, as far as possible, the cells associated with a particular grower (or agent) were coherently grouped and so realistic. We used the citrus distribution of a management control area in east-central Florida (size approximately 35 km x 40 km) for the simulations shown here but note that we have done the same for other control areas with no difference in the qualitative results.

We made the simplifying assumption that the Asian Citrus Psyllid (ACP) populations only develop in grid cells with citrus. In each of these cells, we use an abundance-based population model to describe the population dynamics of ACP. Our model does not account for seasonal variation. The expected lifespan of the ACP is between 30 and 50 days. Therefore, we assumed that a generation of ACP lives for a month and in this time, they may become infected (bacteriliferous) by acquiring CLas from infected trees during feeding and pass that infection on to the healthy trees that they subsequently feed on. We assume that there is no vertical transmission of infection in the population, based on van den Berg et al. and Pelz-Stelinski et al. [39,40] who found little to no vertical transovarial transmission of the bacteria from psyllid parent to offspring. We use a standard discrete population dynamics equation to describe the total number *N* of ACP that emerge in cell *i* in month *t* and survive insecticide spray. This is given by

$$N_{i}(t)=\frac{KM_{i}(t-1)}{\frac{K}{\sigma }+M_{i}(t-1)}[1-\theta_{i}(t)]$$

where *K* is the carrying capacity of the population, *σ* is the number of offspring at low density, *θ* is the efficacy of the insecticide spray applied in month *t*, and *M*_*i*_(*t* − 1) is the total number of ACP in cell *i* and month *t* which is made up of individuals present at *t* and newborns in *i* that did not migrate and the individuals born elsewhere that migrated to *i* between *t* − 1 and *t* (see below). We note that the insecticide spray is applied at *C_f_* months of the year (meaning that *θ*_*i*_ = 0 for 12 − *C*_*f*_ months of the year, with spray months distributed evenly across the year). A proportion of the *N*_*i*_(*t*) ACP are infected with the bacteria giving the total number infected as

$$\tilde{N_{i}}(t)=[1-\text{exp}\left(-\beta \{I_{i}(t)+S_{i}(t)\}\right)]N_{i}(t),$$

where *I*_*i*_(*t*) and *S*_*i*_(*t*) are the numbers of cryptic and symptomatic trees in cell *i* in month *t* respectively and *β* is the rate at which trees pass infection to the ACPs.

Infected ($\tilde{N_{i}}(t)$) and non-bacterialiferous ($\hat{N_{i}}(t)$) populations of ACP disperse according to

$$\tilde{Y_{i}}(t)=\sum_{j=1}^{n}D_{ij}\tilde{N_{j}}(t)$$

$$\hat{Y_{i}}(t)=\sum_{j=1}^{n}D_{ij}\hat{N_{j}}(t)$$

where ${\tilde{Y}}_{i}(t)$ and ${\hat{Y}}_{i}(t)$ represent the populations of infected and non-bacterialiferous psyllid in cell *i* influenced by the flux of individuals from the surrounding cells and so $M_{i}(t)=\tilde{Y_{i}}(t)+\hat{Y_{i}}(t)$, and *D*_*ij*_ is the proportion of psyllids that start in cell *j* and land in cell *i*. We modelled this dispersal with an exponential dispersal kernel. This function is commonly used in insect dispersal models [36,41,42]. The function defines the probability *p_ij_* of ACP starting in cell *j* and landing in cell *i* and therefore ∑_*i*_*p*_*ij*_ = 1. First, we calculate the number of psyllids that remain in their original cell (cell $D_{jj}\tilde{N_{j}}$). We do this by sampling from a binomial distribution with parameters defined by *p*_*jj*_ and the number ACP that could potentially disperse from the cell (i.e. $D_{jj}\tilde{N_{j}}(t)\sim B(\tilde{N_{j}}(t),p_{jj})$). We then move to the cell south of the cell *j* and repeat the process but first adjust the remaining probabilities so that they sum to one (i.e. ∑_*i*,*i*≠*j*_ *p*_*ij*_ = 1) and recalculate the number of ACP still to disperse by subtracting the number that will remain in cell *j* from the total $\tilde{N_{j}}(t)$. In this way working around and outwards, we determined how many ACP landed in each cell each time readjusting the probabilities so that the sum is one and recalculating the number of ACP yet to be assigned to a new cell. The model parameter values (based on [7–10]) are shown in Table 1.

**Table 1:** The parameters for the population dynamics model and the disease model.

Name	Symbol	Value
Carrying capacity of ACP in a cell	*K*	150000 (Based on 300 ACP per tree and 500 trees ha^-1^)
Number of surviving offspring at low density	*σ*	12
Probability infection is passed from tree to ACP	*β*	0.0033*
Probability infection is passed from ACP to tree	*α*	0.0033
Average time (months) for tree to pass from latent to infectious state	1/*γ*	1
Average time (months) for tree to pass from cryptic to symptomatic state	1/*ρ*	6
Dispersal parameter	*λ*	0.00035

*We assumed *α* = *β* and fitted to data on the rate that the number of infected trees increases in a block

The infected psyllids that land in cell *i* in month *t* then infect healthy trees *H*_i_(*t*) in cell *i*

$$E_{i}(t+1)=(1-\gamma )E_{i}(t)+H_{i}(t)(1-e^{-\alpha \tilde{Y_{i}}(t)})$$

$$H_{i}(t+1)=H_{i}(t)e^{-\alpha \tilde{Y_{i}}(t)}$$

where *E*_*i*_(*t*) is the proportion of infected trees in cell *i* in month *t*, *α* is the probability that trees in a cell become infected given that an infected psyllid has fed from them, and 1/γ is the mean time that trees are in a latent stage. Over time, the latent trees become infectious *I*_*i*_(*t*) according to

$$I_{i}(t+1)=\gamma E_{i}(t)+(1-\rho )I_{i}(t)$$

where 1/*ρ* is the mean time that trees are cryptically infectious before passing to the symptomatic state. Infected host tree cells are rare and insignificant epidemiologically before passing to the infectious state, and eventually, the infectious trees become symptomatic *S*_*i*_(*t*) according to

$$S_{i}(t+1)=S_{i}(t)+\rho I_{i}(t).$$

### The behaviour model

In the model, growers face the decision of whether to join an area-wide-control program or not. Their decision is based on their perception of the risk of infection of their orchard by HLB (quantified as, *x*_*r*_ the perceived probability that their orchard will become infected) and their perception of the effectiveness of area-wide control (quantified by, *x*_*c*_ the perceived probability that area-wide control is effective). We quantified these factors for each grower as a value between zero and one, where *x*_*r*_ = 0 represents a perception that there is no risk from HLB and *x*_*c*_ = 0 that they have no faith in area-wide control, and *x*_*r*_ = 1 represents a perception that their orchard will certainly become infected and *x*_*c*_ = 1 that they are convinced that area-wide control is effective. We modelled the dynamics of *x*_*c*_ and *x*_*r*_ over time using opinion dynamics modelling methods [28–31]. Models of opinion dynamics allow us to simulate opinion formation within a group of interacting individuals. The opinion *x*(*i*, *t*) of an individual *i* changes from one-time step *t* to the next by incorporating the opinions of others with their own

$$x(i,t+1)=\sum_{j=1}^{\eta +1}w_{j}x(j,t)$$

where *w*_*j*_ is the weight given to the opinion of individual *j* and $\sum_{j=1}^{\eta +1}w_{j}=1$. The weights can depend on several factors such as the probability that individuals meet each other (which may depend on geographic closeness or some communication network) or the closeness of opinions (individuals with quite different opinions may never be influenced by one another). In our model, an individual *i* interacts with *η* other individuals who are chosen at random with probability proportional to exp(–*d / κ*_*D*_), where *d* is the distance between individuals’ orchards and *κ*_*D*_ is the range parameter. That is to say, that at each time step we select the *η* individuals to interact with grower *i*. The probability that a particular grower *j* is selected to communicate with grower *i* is given by

$$p_{ij}=\frac{\text{exp}(–d_{j}/\kappa D)}{\sum_{k\in \Omega }\text{exp}(–d_{k}/\kappa D)}$$

where Ω represents the population of growers who have not been already selected to interact with grower *i*. We note that for the purposes of this distance calculation we allocated the growers residence to one of the cells they owned at random. Once the *η* growers who communicate with grower *i* are selected we calculate the weights *w*_*j*._ The weights *w*_*j*_ are determined by the closeness of opinion and are proportional to $\text{exp}(-\frac{\left|x(i,t)-x(j,t)\right|}{\kappa_{O}})$ where *κ*_*O*_ is the opinion range parameter. That is to say,

$$w_{j}=\frac{\text{exp}(-\frac{\left|x(i,t)-x(j,t)\right|}{\kappa_{O}})}{\sum_{k=1}^{\eta +1}\text{exp}(-\frac{\left|x(i,t)-x(k,t)\right|}{\kappa_{O}})}.$$

**Fig 2:**
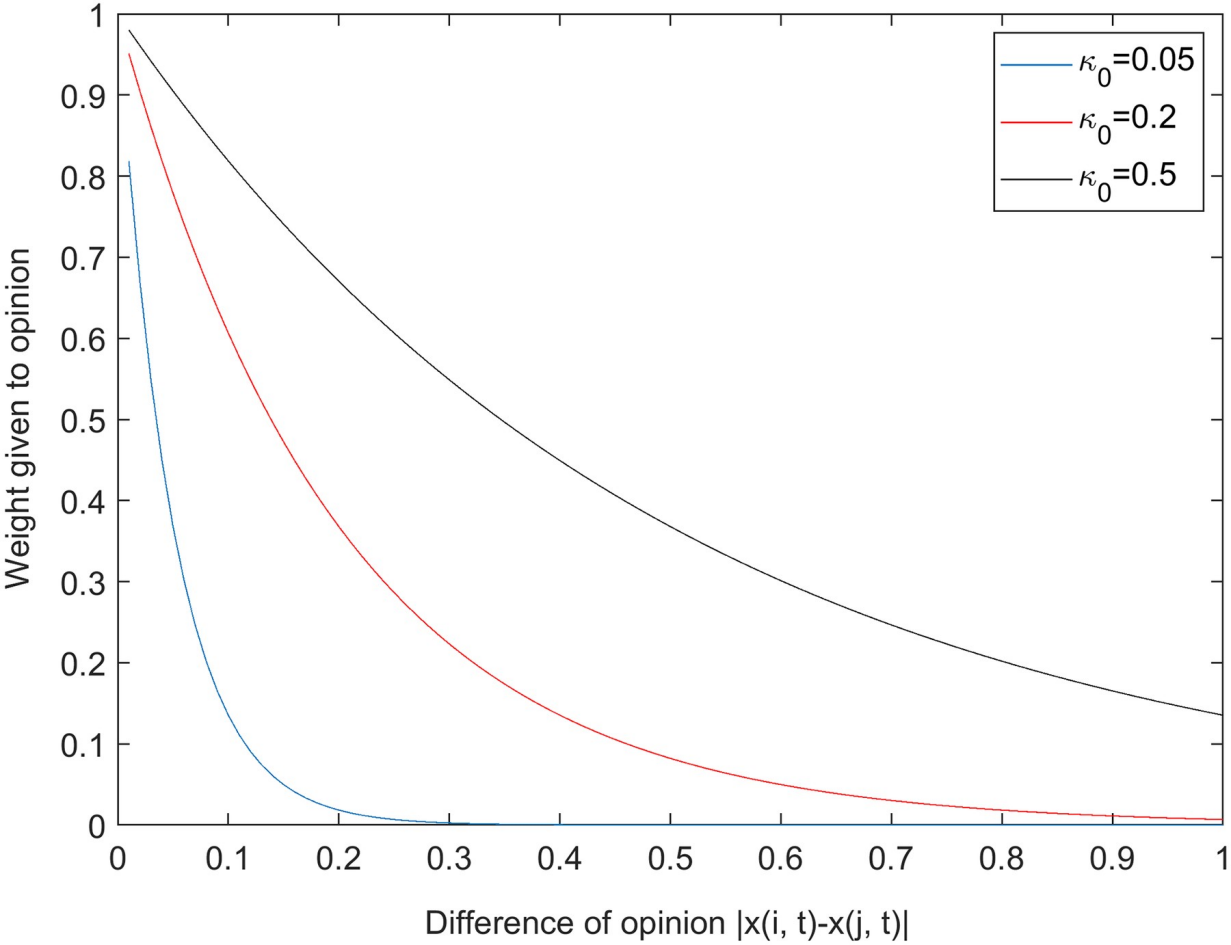
The effect of the parameter *κ*_*O*_ on the weighting of opinion. The relative weight a grower *i* gives to an opinion of grower *j* as a function of the difference in opinion (|*x*(*i*, *t*) − *x*(*j*, *t*)|) as at a given time *t*. All weights are scaled so that $\sum_{j=1}^{\eta +1}w_{j}=1$.

We note that the parameter *w*_*i*_ for grower *i* is largest, meaning an individual’s own opinion has greatest weight. The parameter *κ*_*O*_ alters the weighting of opinions listened to and so convergence of opinion. Larger values of *κ*_*O*_ give more equal weighting across differences in opinion than smaller (see Fig 2), to the extent that if differences of opinion are particularly large they are effectively ignored. This affects opinion consensus and can result in opinion fragmentation (i.e. the phenomena where the opinions of agents will never converge to one value, see [30] for more discussion and illustrations). We also included the influence of extension agents on the opinions of the growers. In the model, extension agents disseminate information on HLB control to all growers at a frequency of *E*_*f*_ times per year. This increase the growers’ perceptions of the risk of becoming infected by HLB (*x*_*r*_) and their belief that area-wide control is effective (*x*_*c*_) by a given amount *E*_*I*_. That is to say

$$x(i,t+1)=max(\sum_{j=1}^{\eta +1}w_{j}x(j,t)+E_{I}\delta (mod\{t,\frac{12}{Ef}\}),1),$$

where *δ* is an impulse function that takes the value of *δ*(0) = 1, and zero elsewhere. If a grower observes more than 0.2% of trees with infection in a cell that they manage (which equates to a whole tree) then *x*_*r*_ becomes one. Similarly, if a grower joined an area-wide control program at least *h*_*m*_ months ago but still observes an average increase in disease greater than 1% across his orchards then *x*_*c*_ reduces by a factor of *δ*. That is to say, if grower *i* (who owns Λ cells) has been part of the area-wide control program for at least *h*_*m*_ months and observes

$$\frac{1}{\Lambda_{i}}\sum_{k=1}^{\Lambda_{i}}\left\{S_{k}(t)-S_{k}(t-1)\right\}>1$$

then the grower’s belief that area-wide control is effective (*x*_*c*_) will reduce by *δ*.

In the model, growers join the area-wide-control program if *x*_*c*_ and *x*_*r*_ exceed given thresholds. An insecticide spray is applied a fixed number of times per year to all orchards managed by individuals who have joined the area-wide-control program.

### Implementing the model

We assigned to each of the parameters in the opinion dynamic model a set of values that, according to our experts, spanned realistic ranges (Table 2). Similarly, we assigned sets of values to the parameters that described the efficacy of the insecticide and the number of insecticide applications that were undertaken each year under area-wide control. The parameter values of the epidemiological model remained at the default settings as these parameter values are, as explained, relatively well known. Next, a large series of simulations were done using all combinations of parameter values.

**Table 2:** The parameters for the grower-decision and the control models, with the values used in our analysis. The numbers in bold were used in the simulations except when otherwise stated.

Name	Symbol	Parameter values explored
Number of individuals that communicate. This parameter affects the rate of convergence of opinion. If only few individuals interact in a time-step the convergence will take longer.	*η*	10, **60**, 120
The range parameter determining the probability two growers communicate (km). This parameter affects the rate of spatial spread of opinion formulation. If the range parameter is small then spatial spread will be somewhat slower.	*κ*_*D*_	1, **3**, 12
The range parameter determining the weighting of opinions based on closeness of opinion. This affects how individuals weigh the opinions of others given how close that opinion is to the one the individual currently holds. Larger values of *κ*_*O*_ give a more equal weight.	*κ*_*O*_	**0.05**, 0.2, 0.5
Frequency that information is disseminated by extension agents (number of times per year).	*E*_*f*_	**0**, 3, 6, 12
Impact of information from extension agents. The greater the value the more impact the extension agent has on the growers’ opinions.	*E*_*I*_	**0.2**, 0.4, 0.6
History of control (months). If the grower joined an area-wide control program at least *h*_*m*_ months ago but still observes an increase in disease greater than 1% across his orchards they loose faith in area-wide control (i.e. *x*_*c*_ decreases by *δ*).	*h*_*m*_	**2**, 6
Reduction in belief if infection is observed despite control being applied	*δ*	**0.4**
Mean initial belief in control across the population of growers	*μ*_*c*_	**0.2**, 0.5, 0.8
Variance of initial belief in control across the population of growers	$\sigma_{c}^{2}$	0.01, 0.05, **0.1**
Mean initial risk perception across the population of growers	*μ*_*r*_	0.2, **0.5**, 0.8
Variance of initial risk perception across the population of growers	$\sigma_{r}^{2}$	0.01, 0.05, **0.1**
Frequency of control–the number of sprays per year.	*C*_*f*_	6, **12**
Kill rate	*θ*	0.7, **0.94**

## Results

**Fig 3:**
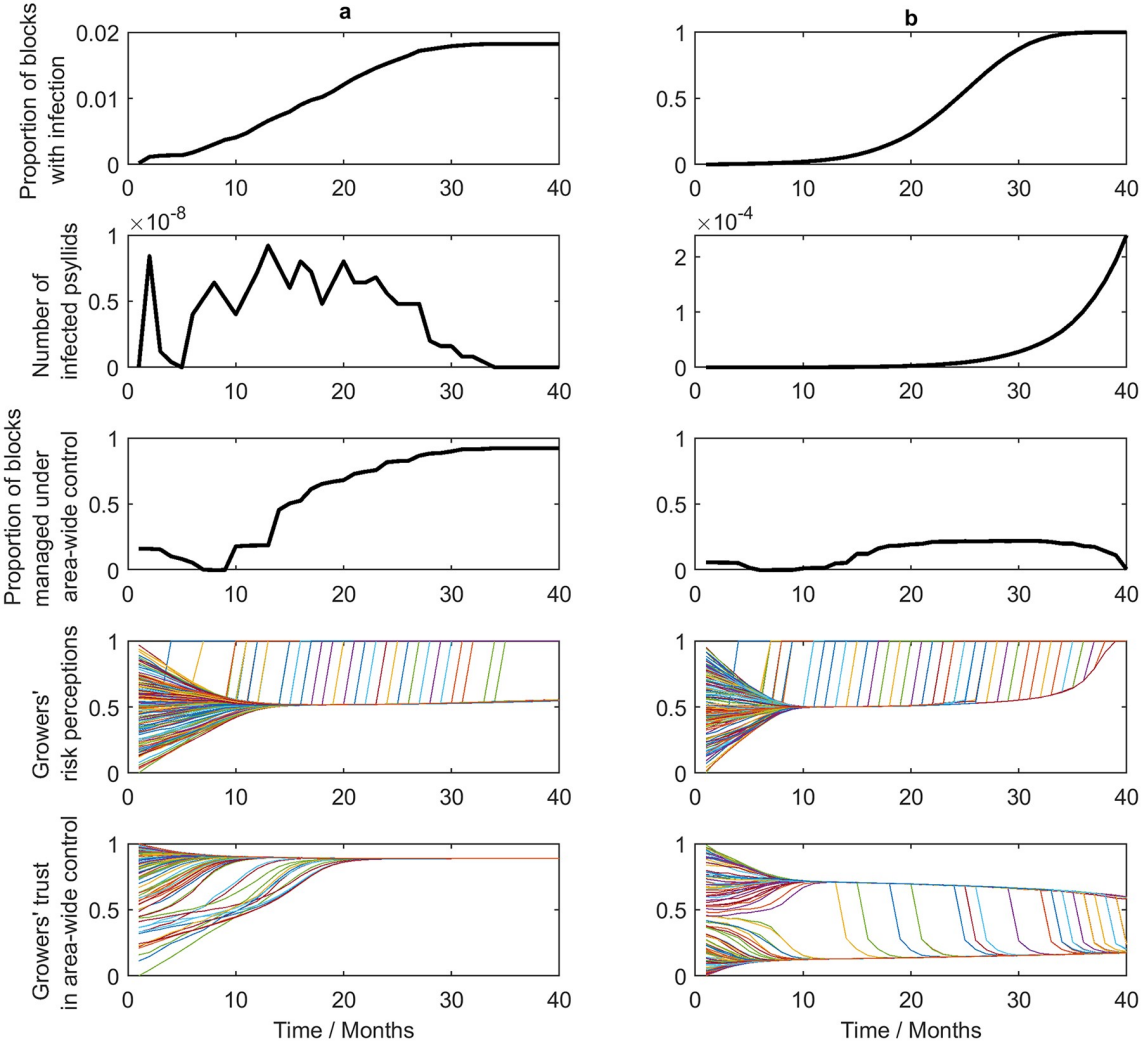
**(a) A simulated scenario where control of HLB is successful**. The evolution of risk perception and trust in control is shown for each grower in the region (each growers perception is represented by a coloured line). Risk sharply increases when growers observe disease in their orchards and then quickly persuade their neighbours the disease is a serious threat and to trust in area-wide control. (b) **A simulated scenario where control of HLB fails**. Grower uptake of control is not rapid enough to control the disease and so the disease becomes endemic and proliferates. Growers who have joined an area-wide control program observe that it is not working and drop out. We note the model has a monthly timestep.

There are broadly two types of outcome found in our simulations. Firstly (Fig 3a) control success. In this outcome scenario, the number of orchard blocks infected and the density of bacterialiferous (CLas infected) psyllids initially increase. This, in turn, increases the risk perception of growers to such an extent that they join the area-wide control program. As growers join the control programme, the density of bacterialiferous psyllids decreases and the number of infected orchard blocks does not further increase, leading to an epidemic under control: Control success. The second possibility (Fig 3b) is control failure. Initially, growers start joining the area wide control scheme, but their trust in control is compromised because after joining the area wide control scheme their orchards become infected. This stimulates them to drop out of the area-wide control program, consequently the epidemic grows rapidly and eventually most orchards become infected: Control failure.

The control success and the control failure shown in Fig 3 resulted from exactly the same set of parameter values. The only difference between the two simulations is the mean initial risk perception and the mean initial trust in control of the growers (‘mean’ because each grower has initial values for risk perception and trust in area-wide control that are drawn from a beta distribution with a defined mean and variance). It must be said that the simulations shown were chosen from a set of runs, where due to the stochastic nature of the model, some runs show control success while others control failure. We consider the question, “Is this result caused by the particular set of parameter values selected or is it a more general phenomenon?” We did a sensitivity analysis to establish which factors in our opinion dynamics model were most important for control success. We also did further simulations and calculated the probability of control success or control failure for a range of mean initial risk perception and main initial trust in the control options of the growers (see below).

### Sensitivity analysis

Model parameters for the epidemiological model were based on published information on the epidemiology of HLB and from published models [35–38]. For the opinion dynamics part of the model there are no parameter values known to quantify the effects of interactions of growers on their opinion, no quantitative information is known about the effect of consultants, extension workers and researcher on grower opinion, nor is anything known about the level of risk and level of trust needed for a grower to join the area-wide control program. Therefore, we used expert knowledge elicitation to obtain plausible ranges for these parameters. To this end, ranges of parameter values for the opinion dynamics model were provided to us by experts (one of them is the second author). The initial risk perception and initial trust in the control for each individual grower were sampled from beta distributions with defined mean and variance (see Methods).

We did a sensitivity analysis to establish which factors in our model were most important for control success. Our emphasis is on factors related to control efficacy and grower behaviour. For this reason, (and because the parameters’ values for the epidemiological model are relatively well known) the parameter values of the epidemiological model remained at the default settings and we only explored the model sensitivity to the parameters related to control efficacy and behaviour (see Methods, Table 1 for details of the 11 parameters explored and the sets of values used).

We analysed the simulation results using ANOVA with up to three-way interactions to identify the most important factors for controlling the epidemic (measured as the proportion of cells infected at the end of 36 months). In the ANOVA, parameters were treated as factors, while specific parameter values (4 for one of the parameters, 3 for 8 of the parameters and 2 for 3 of the parameters) were treated as levels (Table 1). We could not treat all 11 parameters as factors at once because we had no replication (i.e. we ran each combination of parameter values only once). Therefore, we used combinations of eight parameters at a time as factors and compared the percentage variance accounted for to determine which combination of factors best explained the proportion of infected cells. That is to say, we considered 495 statistical models (*C*_12,8_ “12 choose 8”) to see which combination of eight parameters accounted for the most variation. Once the best model was selected, we used the F-probability to order the importance of the eight factors used in the final selected model.

In total 209952 (= 4 × 3^8^ × 2^3^) simulations were done to explore all combinations of the parameters. The model that accounted for the most variation in the response variable (the proportion of cells infected, Ω) was

$$\Omega =\kappa_{0}\times E_{F}\times E_{I}\times \mu_{c}\times \mu_{r}\times \sigma_{c}^{2}\times C_{f}\times \theta$$

where each parameter is described in Table 3.

**Table 3:** The ANOVA table reporting main effects only.

Source of variation	Degrees of freedom	Sum of squares	Mean squares	Variance ratio	F pr.
Opinion range (*κ*_*0*_)	2	43.77	21.89	450.45	< .001
Extension information freq. Number of interactions per year (*E*_*F*_)	3	3158	1053	21664.67	< .001
Extension information effect (*E*_*I*_)	2	7.345	3.673	75.59	< .001
Mean initial risk perception across the population of growers (*μ*_*r*_)	2	8.083	4.041	83.18	< .001
Mean initial belief in control across the population of growers (*μ*_*c*_)	2	1309	654.7	13475.39	< .001
Variance in belief ($\sigma_{c}^{2}$)	2	13.02	6.512	134.03	< .001
Freq. of spray (number of sprays per year) (*C*_*f*_)	1	9166	9166	188600	< .001
Spray efficacy (*θ*)	1	7854	7854	161700	< .001
Residual	209936	10200	0.04859		
Total	209951	31760			

**Fig 4:**
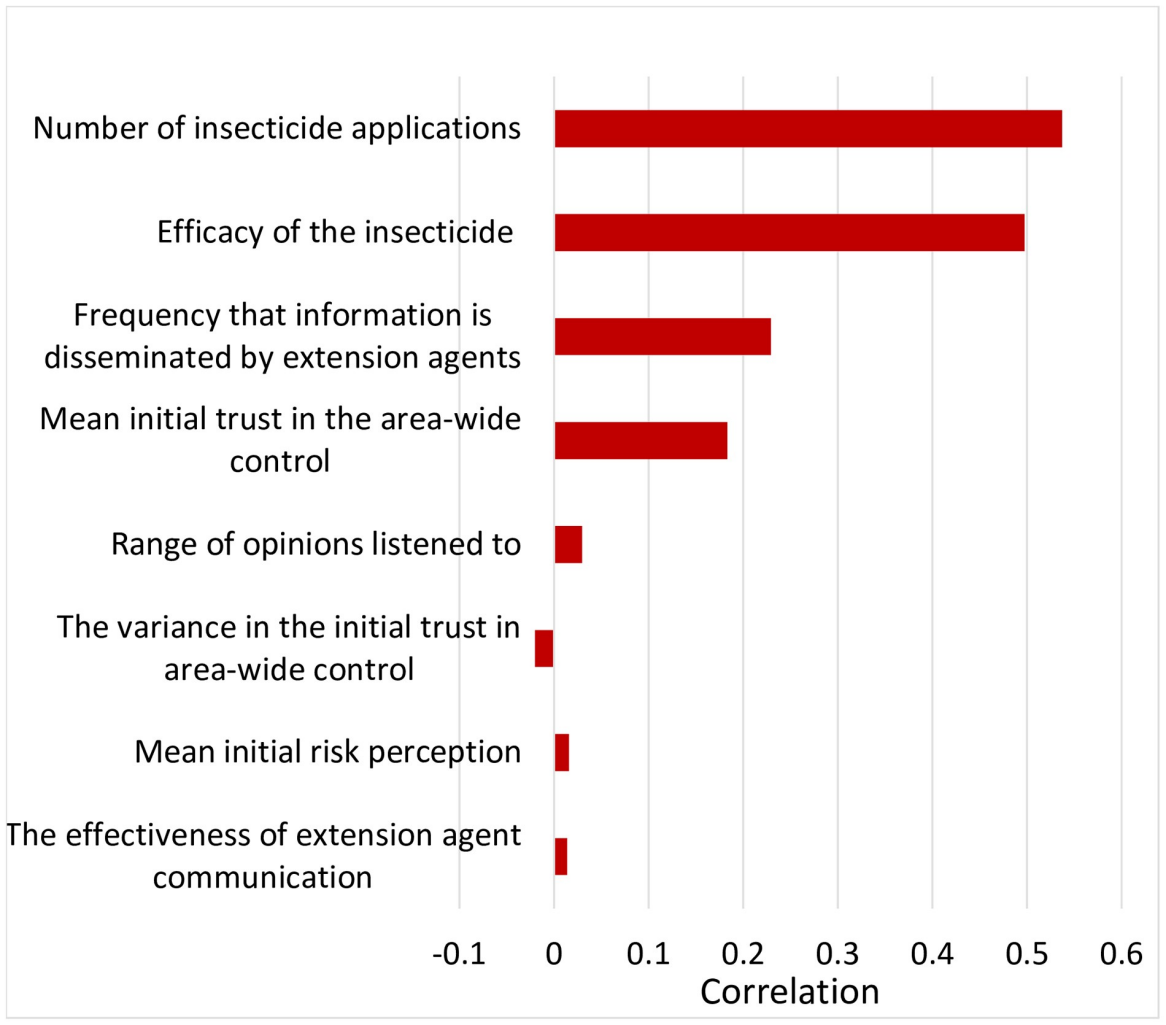
Tornado graph showing the correlation between each variable and the proportion of cells infected.

All of these factors and many of their two and three-way interactions were highly significant <0.001 (Table 3), which can be attributed to the large size of the data set. These are shown in Fig 4 in order of highest to lowest importance.

**Fig 5:**
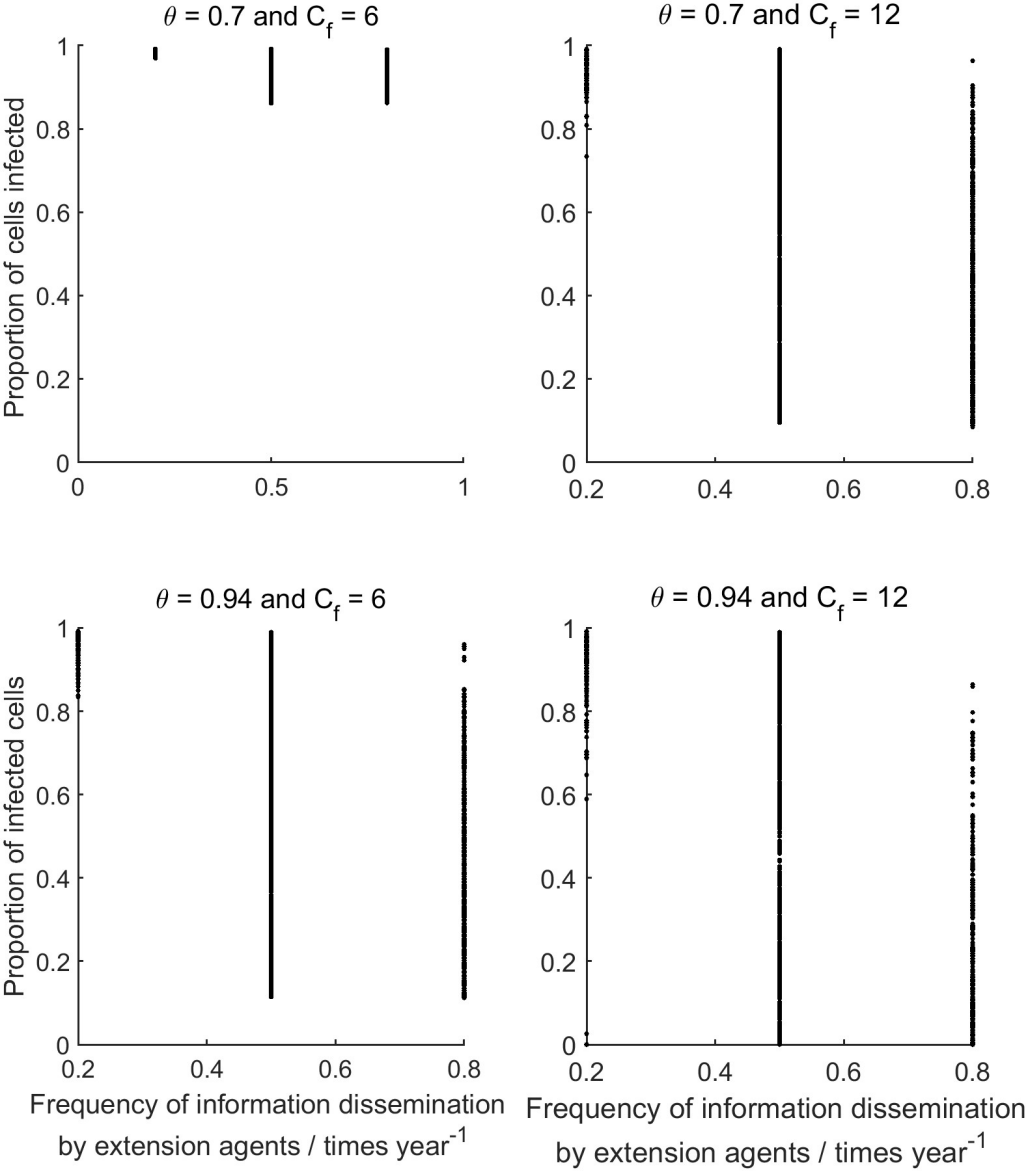
The results of the simulations. The proportion of cells (1 ha areas) infected at the end of the simulation is plotted against the frequency at which information is disseminated by extension agents for each combination of spray efficacy (*θ*) and spray frequency (*C*_*f*_). All other parameters values were varied as described in Table 2 resulting in the ranges of outcomes observed for each combination of *θ* and *C*_*f*_.

**Fig 6:**
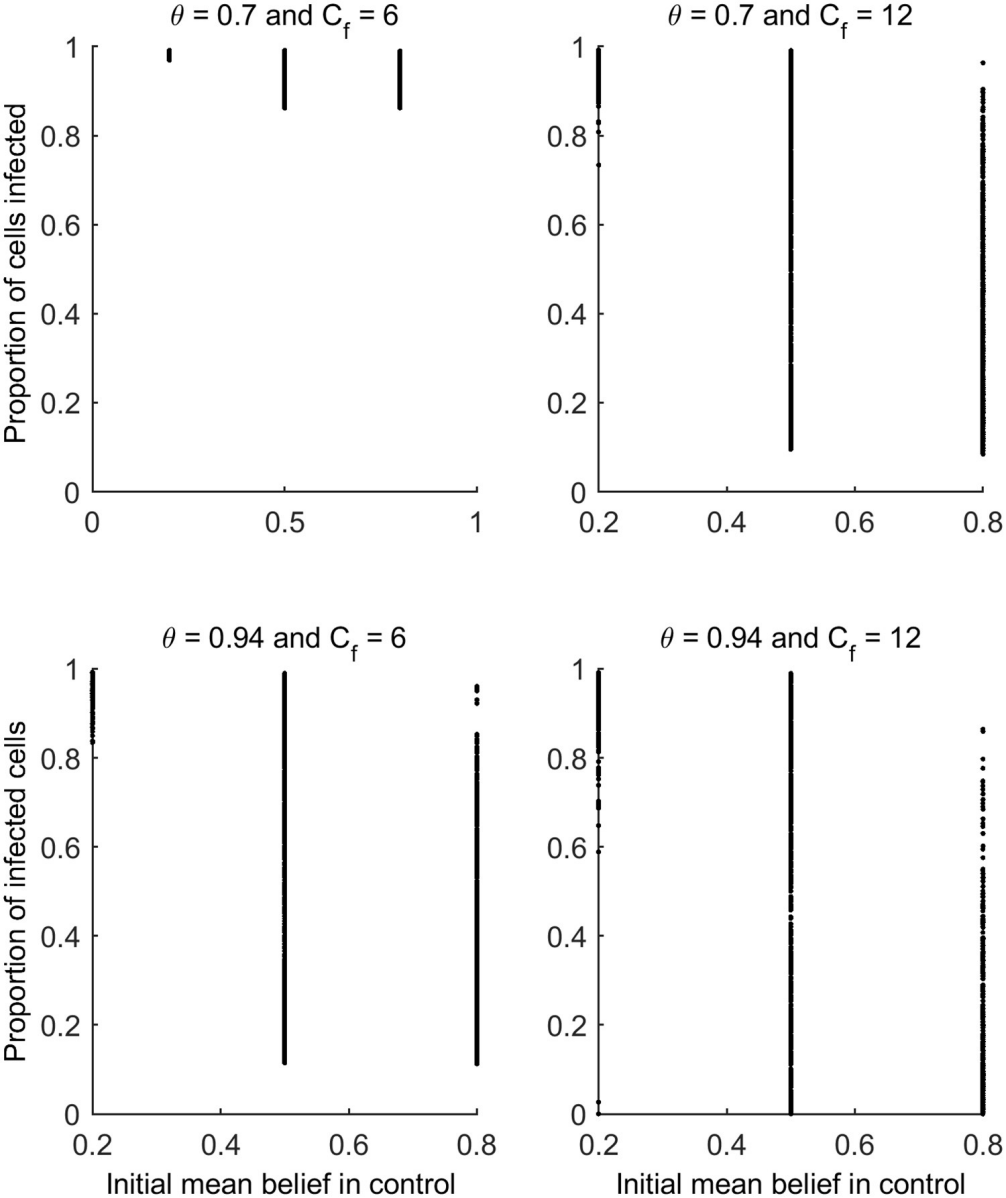
The results of the simulations. The proportion of cells (1-ha areas) infected at the end of the simulation is plotted against the initial belief in the control method for each combination of spray efficacy (*θ*) and spray frequency (*C*_*f*_). On all simulations the frequency information was disseminated was set to zero (*E*_*f*_ = 0). All other parameters values were varied as described in Table 2 resulting in the ranges of outcomes observed for each combination of *θ* and *C*_*f*_.

**Fig 7:**
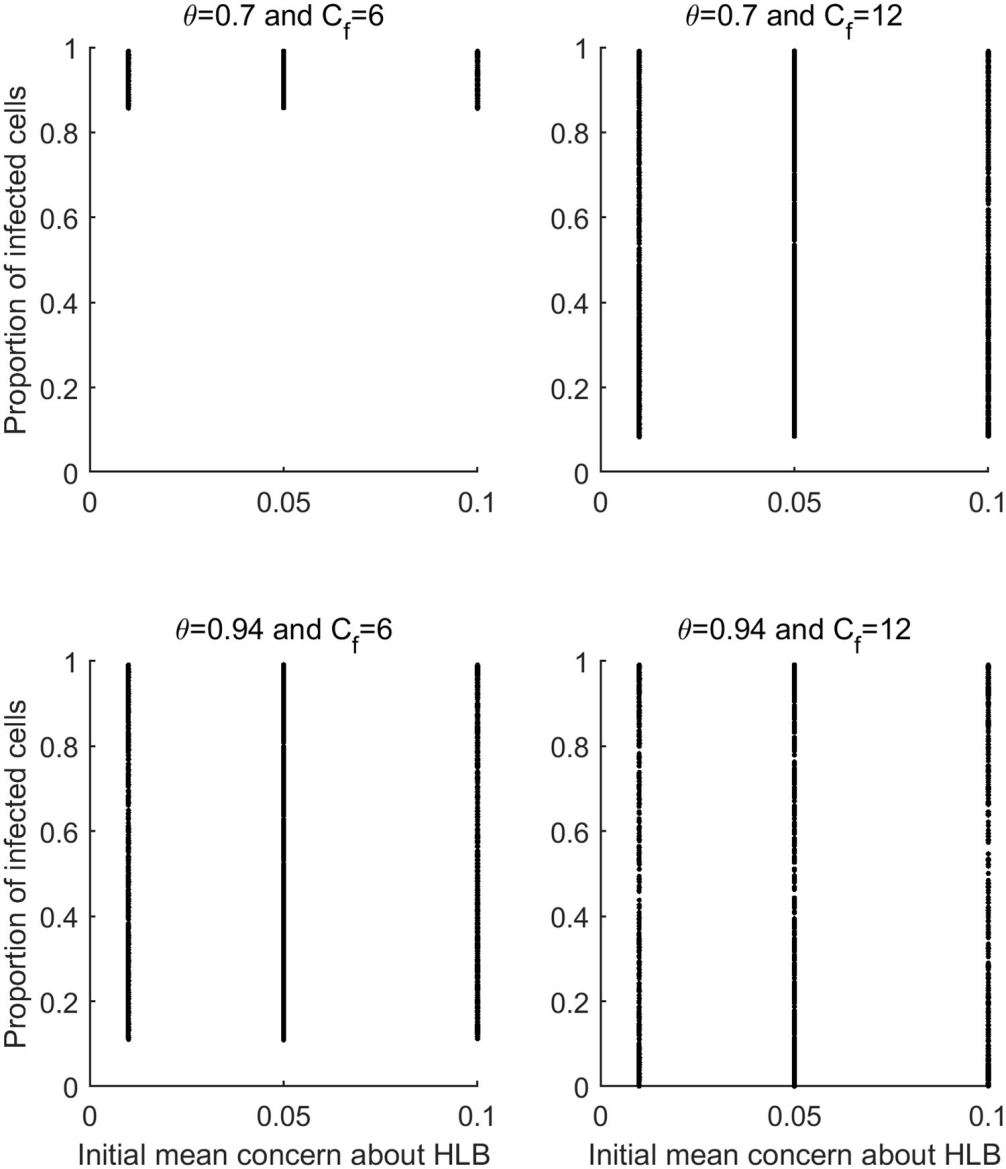
The results of the simulations. The proportion of cells (1-ha areas) infected at the end of the simulation is plotted against the initial risk perception about HLB for each combination of spray efficacy (*θ*) and spray frequency (*C*_*f*_) on all simulations the frequency information was disseminated was set to zero (*E*_*f*_ = 0). All other parameters values were varied as described in Table 2 resulting in the ranges of outcomes observed for each combination of *θ* and *C*_*f*_.

Further exploration showed that if the efficacy of the insecticide, *θ* is poor (either because the insecticide is ineffective or because it is applied too infrequently) then control success cannot be achieved (Figs 5 and 6). If the insecticide is effective, however, frequent and effective contact with extension services can substantially increase the probability of control success (Fig 5). Initial trust in the control method also has a strong effect (Fig 6) compared with, for example, the initial risk perception of the growers (Fig 7).

**Fig 8:**
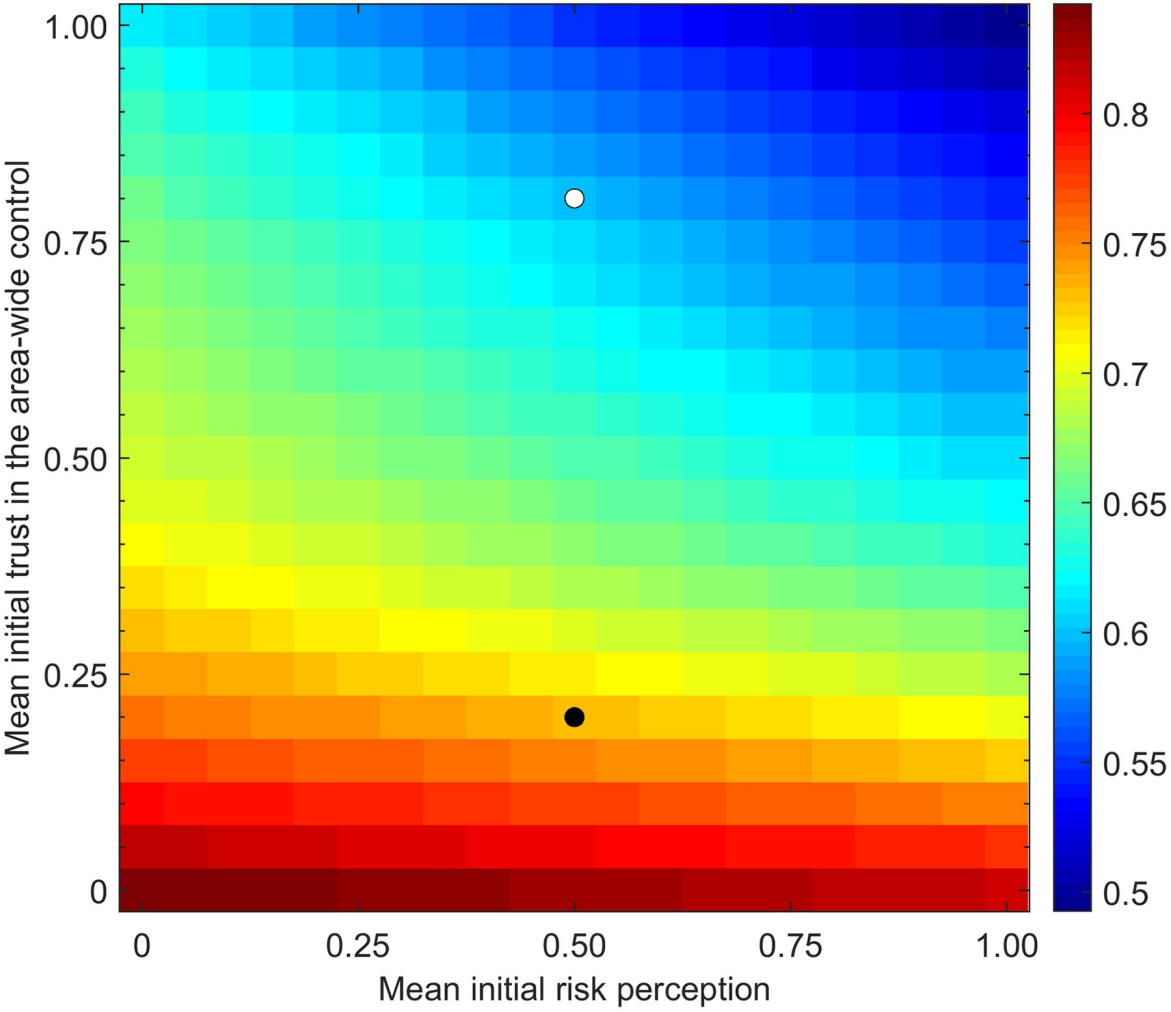
The probability of control failure considering grower initial risk perception versus trust in area-wide control. The white disc relates to the simulation shown in Fig 3a and the black disc to the simulation shown in Fig 3b.

We did further simulations and calculated the probability of control success or control failure for a range of mean initial risk perception and main initial trust in the control options of the growers (Fig 8). This confirms and illustrates that the mean initial trust in the control options is a far more important factor in the success of the disease control campaign than the mean initial risk perception.

## Discussion

To our knowledge, plant-epidemiological models have never been coupled with opinion dynamic models. We show here how an opinion dynamic model can be intuitively coupled to an epidemiological model. The key aspect is that the opinion of a given grower on risk and trust in control (which are influenced by the opinions of other individuals and the direct observation of the epidemic) determine the probability that the individual will join the area-wide-control scheme. In turn, the control affects the course of the epidemic. Opinions are key driving forces behind human decision making and so it is essential that these are accounted for when considering how to maximize the potential impact of voluntary disease control campaigns such as those associated with the control of HLB. We have shown that coupling an epidemiological model with an opinion dynamic model can give insight into these sorts of systems.

Clearly, and intuitively, the efficacy of the control program, i.e., the insecticide kill rate and the number of applications, is the most important factor in the success of an HLB control campaign. Of the opinion-dynamic parameters, we have shown that for HLB aiming to inform growers about the effectiveness of the regionally coordinated control actions and the efficacy of the insecticide program may be of greatest importance. This coincides with the wider social science literature where it is reported that grower engagement with advice and support networks plays a critical role in the take-up of participatory activities such as regional pest control or agri-environmental schemes [43–45]. This is thought to be because these networks provide a valuable source of information and help to engender a sense of shared responsibility. The extent to which engagement can shape attitudes towards belief is dependent on the length and frequency of engagement ([7] and references within): if contact between extension services and growers becomes infrequent then important scientific messages can become forgotten or diluted. This agrees with the findings of our model.

The initial trust in the control options is also shown to be of key importance, just as in the simulations shown Figs 3 and 8. The most surprising finding is that the initial risk perception plays a relatively unimportant role in determining the success of an HLB control campaign. Further analysis of this phenomenon showed that the reason for this is found in the effect of the direct disease observations on the risk perception. When the epidemic starts to infect more and more orchards, growers form opinions from ‘direct’ observation concerning the chances that their orchards will also become infected. These direct observations start to override risk perception dynamics due to the ‘indirect’ opinions derived from other growers. This increased risk perception does not apply to the trust in the control option. The social dynamics with advisors increasing the trust in control is still strongly, but negatively affected when growers see the control failing. In practice, perceptions of the level of risk of disease have been shown to be connected with the proximity of disease [7]. Therefore, it is important that growers are given frequent and accurate updates on the location and intensity of disease outbreaks as well as clear information on appropriate control measures.

The range of opinions that growers pay attention to was also on our list of important social factors. In practice, this relates to how willing growers are to adapt their opinions to views quite different from their own. If growers are well connected, then it is more likely that their opinions will evolve over time as they will encounter ranges of opinions that may slowly persuade them. This reflects the phenomenon that growers who are more isolated in opinion or not well networked tend to be more difficult to engage as they lack information and are reported to be potentially more immune to influences from their peers [46].

In many information programs aimed at informing and preparing the public, or a professional group of a possible infectious disease, there often is much emphasis on the risks the disease poses. Our research questions whether that is a necessary approach, especially in the light of the potential loss of trust with the public if the epidemic does not actually take place, as was the case for the official swine flu warning in 2009 [34]. We note however that we have shown this for HLB and it remains to be investigated whether this holds more generally or if the emphasis of information campaigns should be decided on a case by case basis.

## Supporting information

S1 Data From the simulation runs. (ZIP)
